# Endogenous Ceramide Contributes to the Transcytosis of oxLDL across Endothelial Cells and Promotes Its Subendothelial Retention in Vascular Wall

**DOI:** 10.1155/2014/823071

**Published:** 2014-04-10

**Authors:** Wenjing Li, Xiaoyan Yang, Shasha Xing, Fang Bian, Wanjing Yao, Xiangli Bai, Tao Zheng, Guangjie Wu, Si Jin

**Affiliations:** ^1^Department of Pharmacology, Tongji Medical College, Huazhong University of Science and Technology, Wuhan 430030, China; ^2^The Key Laboratory of Drug Target Research and Pharmacodynamic Evaluation of Hubei Province, Wuhan 430030, China

## Abstract

Oxidized low density of lipoprotein (oxLDL) is the major lipid found in atherosclerotic lesion and elevated plasma oxLDL is recognized to be a risk factor of atherosclerosis. Whether plasma oxLDL could be transported across endothelial cells and initiate atherosclerotic changes remains unknown. In an established *in vitro* cellular transcytosis model, the present study found that oxLDL could traffic across vascular endothelial cells and further that the regulation of endogenous ceramide production by ceramide metabolizing enzyme inhibitors significantly altered the transcytosis of oxLDL across endothelial cells. It was found that acid sphingomyelinase inhibitor, desipramine (DES), and *de novo* ceramide synthesis inhibitor, myriocin (MYR), both decreasing the endogenous ceramide production, significantly inhibited the transcytosis of oxLDL. Ceramidase inhibitor, N-oleoylethanolamine (NOE), and sphingomyelin synthase inhibitor, O-Tricyclo[5.2.1.02,6]dec-9-yl dithiocarbonate potassium salt (D609), both increasing the endogenous ceramide production, significantly upregulated the transcytosis of oxLDL. *In vivo*, injection of fluorescence labeled oxLDL into mice body also predisposed to the subendothelial retention of these oxidized lipids. The observations provided in the present study demonstrate that endogenous ceramide contributes to the transcytosis of oxLDL across endothelial cells and promotes the initiating step of atherosclerosis—the subendothelial retention of lipids in vascular wall.

## 1. Introduction

Atherosclerosis (AS) is the pathological basis of cerebro- and cardiovascular diseases, which are the leading causes of death in the elderly people [1]. The pathogenesis of AS is far from fully understood. In recent years, more and more evidences show that the initiating step of AS is likely to be the subendothelial retention of lipoprotein in intima, so called “response to retention” hypothesis [2–5], which highlights the role of accumulation of apoB-containing lipoproteins (mainly low density lipoprotein, LDL) in the subendothelial space, as well as extracellular matrix adhesion molecules, and so forth in the pathogenesis of AS.

To be deposited in the vascular intima, lipoproteins must pass through the barrier provided by vascular endothelium. The gap between the vascular endothelial cells is known to be about 3–6 nm in diameter, only allowing water, inorganic salts, and a handful of small proteins to pass through. Such macromolecule molecules as LDL in a diameter of about 20–30 nm are not generally able to pass through the endothelial barrier [6–8].

Recent studies have found LDL traffic across endothelial barrier mainly through transcytosis [9], which refers to a process of protein-rich particles passing through polar cells (such as endothelial cells, epithelial cells, etc.) by receptor or nonreceptor mediated mechanisms. LDL can be endocytosed by vascular endothelial cells in the lumen side and then exocytosed to the basolateral side [9]. A large number of studies have documented that the major LDL accumulated in AS plaques is in the oxidative modified form, namely, oxidized low density lipoprotein, oxLDL. Currently, oxLDL is recognized as the major causative factor of AS [10, 11]. Plasma oxLDL levels were significantly increased in AS patients and have been considered as indicators of the early screening and diagnosis of coronary heart disease [12, 13]. Whether plasma oxLDL can pass through endothelial cells by transcytosis and further stick to intima to initiate the incidence of AS remains unknown.

In recent years, the role of lipid rafts (LRs) in the transmembrane transport of macromolecules has attracted much more attention [14–17]. Lipid rafts are membrane domains rich in sphingolipids and cholesterol [18, 19]. Endothelial caveolae used for LDL transcytosis is a specialized membrane raft domain [15]. Membrane fusion between vesicle membranes and sarcoplasmic membranes due to the formation of macromolecular complexes, such as t-SNARE and v-SNARE, is also dependent on the platform formed by fusion of membrane rafts [9]. Ceramide, the backbone of sphingolipids, is thought to participate in the development of atherosclerosis [20, 21]. Ceramide can be generated from sphingomyelin through activation of sphingomyelinase (SMases) or from the* de novo* pathway. Also, ceramide can be synthesized to sphingomyelin through activation of sphingomyelin synthase (SMS) or degraded into sphingosine by ceramidase, respectively. Inhibitors involved in ceramide metabolism commonly including acid sphingomyelinase (ASM) inhibitor, desipramine [22–24],* de novo* ceramide synthesis inhibitor, myriocin [25], ceramidase inhibitor (NOE) [26, 27], and sphingomyelin synthase inhibitor (D609) [28, 29].

Upon the stimulation of endogenous and exogenous factors, the sphingolipid (sphingomyelin) in endothelial cell membrane rafts undergoes hydrolysis by acid sphingomyelinase, releasing the hydrophilic phosphocholine group and generating hydrophobic product, ceramide [15]. The existence of intermolecular hydrogen bonds provides strong driving force for ceramide to fuse simultaneously. Through the integration of ceramide, many small membrane rafts can cluster together into larger microdomains, which provide signaling platforms for the interaction of transmembrane signal transduction [30–32]. Recent studies have also found that the ceramide produced by membrane rafts plays key roles in pathogen invasion into host cells, such as* Pseudomonas aeruginosa* [33–35]. In addition, ceramide can trigger and promote the exocytosis of Weibel-Palade bodies in endothelial cells [23].

Given the multiple origins of cellular ceramide, the current study aims to determine the roles of ceramide from different origins in mediating the transcytosis of oxLDL across the vascular endothelial cells and how these transcytosed oxLDL particles further promote AS changes in vascular walls.

## 2. Methods

### 2.1. Isolation and Culture of Human Umbilical Vein Endothelial Cells (HUVECs)

The collection of human umbilical cords was approved by the Ethics Committee of Tongji Medical College, Huazhong University of Science and Technology (Wuhan, China), and conducted in accordance with the Declaration of Helsinki (2008). Primary HUVECs isolated from 0.01% EDTA-0.25% trypsin digested newborn umbilical cord were cultured in ECM (ScienCell) supplemented with 5% fetal bovine serum (FBS), 100 U/mL penicillin, 100 U/mL streptomycin, and 30 *μ*g/mL endothelial cell growth supplement (ECGS) at 37°C under 5% CO_2_ in a humidified atmosphere. For subculture, cells were harvested with 0.25% trypsin without EDTA when 80%~90% confluent. Before experiments, ECM was replaced with OPTI-MEM (Gibco) without FBS. All studies were performed using HUVECs of 2 to 8 passages [36–38].

### 2.2. Immunocytochemistry

After incubated with inhibitors (MYR, DES, D609, and NOE), respectively, for 12 h, HUVECs grown on gelatin-coated coverslips were fixed in 4% formaldehyde for 10 min and then washed in PBS for three times. Cells were stained with an anti-ceramide IgM antibody (Alexis, 1 : 100) for 1 h at 37°C and followed by CY3-conjugated goat anti-mouse secondary antibody (Bioss, 1 : 100) for another 2 h at room temperature [39–41]. Images were acquired using a custom-configured fluorescence microscope (Olympus FV500) [42]. The integrated fluorescence intensities were measured using the Image-Pro Plus software and normalized to the number of cells.

### 2.3. Intracellular Ceramide Extraction and Quantitation

Ceramide was extracted and quantified by HPLC-MS/MS (Thermo, LCQ DECA XP^plus^) according to the principles described previously [43–46]. For extraction of cellular lipids, cells were lysed with distilled water and homogenized by sonication after incubation with inhibitors for 12 h. Protein concentrations were measured and the equal amounts of protein (500 *μ*g) were adjusted to the volume of 800 *μ*L in 1 M NaCl. C_12_-ceramide (10 ng) was added to lysates as an internal standard and the resulting samples were extracted with chloroform/methanol (1 : 2) 3 mL for 3 h. Samples were then centrifuged at 3000 g, 5 min. Supernatants were transferred to the other tubes within CCL_4_ and 1 M NaCl 1 mL, respectively. After centrifugation, the lower organic phase was obtained and evaporated to near dryness under a gentle stream of dry N_2_. Meantime, samples were reconstituted by 100 *μ*L methanol to measure ceramides C_14_, C_16_, C_24:1_, and C_24_ by HPLC-MS/MS. The levels of each ceramide species were determined by their relative abundance normalized to C_12_-ceramide and the gross of ceramides was quantified based on the standard curve which was constructed on ceramide standards (Avanti). The gross of these ceramides was used for statistics.

### 2.4. Cellular Uptake of oxLDL

oxLDL was labeled with fluorescein isothiocyanate (FITC; Biosharp) by a minor modification of a previously described method [47, 48]. In brief, oxLDL and FITC were mixed and incubated at 37°C for 2 h and then unbound FITC was removed by dialysis against PBS for 72 h at 4°C. Finally, FITC-oxLDL was stored at 4°C in the dark for further use. All procedures were performed in the dark. Cells were seeded on gelatin-coated glass coverslips in 24-well culture dishes and incubated at 37°C and 5% CO_2_. After being treated with the above inhibitors, respectively, for 9 h, cells were then incubated with 50 *μ*g/mL FITC-oxLDL for 3 h. Images were obtained with a fluorescence microscope using a 40x objective. The integrated fluorescence intensities were measured using the Image-Pro Plus software and normalized to the number of cells.

### 2.5. oxLDL Retention in Isolated Umbilical Venous Wall

In a mixture of oxygen (95% O_2_ and 5% CO_2_) condition, the human umbilical venous rings were incubated with 50 *μ*g/mL FITC-oxLDL and various inhibitors at 37°C for 3 h [47]. Then the tissues were frozen and sliced into thin sections of 8 *μ*m with a freezing microtome (Leica CM1900) and further stained with DAPI. For each optical section, the space above the basilar membrane was defined as the region of interest (ROI). The fluorescence intensity was quantitated using Image-Pro Plus software. A weighted analysis was performed by first determining the area of fluorescence within the ROI of each optical section for three fluorescence intensity value ranges as follows: 160 to 190, 190 to 210, and 210 to 230. These three area measurements were then multiplied by 1, 3, or 5, respectively, to give greater weight to areas of highest intensity [49]. These weighted values were then summed for each optical section and divided by the area of ROI.

### 2.6. oxLDL Transcytosis

As described previously, the amount of oxLDL transcytosis was measured by a nonradioactive method* in vitro* [47, 48]. HUVECs were seeded on polyester membrane of costar transwell (6.5 mm diameter and 0.4 *μ*m pore size) to form integrated cell monolayer. The integrity of cell monolayer was tested by a method described previously [50]. Two inserts of cell monolayers with equal integrity were divided into the same group: the noncompetitive insert and the competitive insert, respectively. Inhibitors were added to each group for 9 h. And then, the noncompetitive insert was incubated with 50 *μ*g/mL FITC-oxLDL to determine the total amount of transendothelial oxLDL; paracellular transport was determined by incubation with 50 *μ*g/mL FITC-oxLDL and 6-fold excess of unlabeled oxLDL (300 *μ*g/mL) in competitive insert. After 3 h, samples were then collected from the outer chambers and further dialyzed against PBS to remove the free FITC. The relative fluorescence was measured via a fluorescence spectrophotometer (Tecan, Infinite F200PRO) with excitation and emission wavelengths of 490 nm and 520 nm, respectively. Meanwhile, background fluorescence determined by serum-free Opti-MEM was subtracted from the value of each sample. The amount of oxLDL transcytosis is the difference between the fluorescent intensity of the noncompetitive insert and the competitive insert.

### 2.7. Subendothelial oxLDL Retention* In Vivo*

Animals were treated in accordance with the guide for the Care and Use of Laboratory Animals published by the US National Institutes of Health and approved by the local animal care committee. Healthy C57BL/6J mice (18–20 g) were purchased from the Center of Experimental Animals (Tongji Medical College, Huazhong University of Science and Technology, China) and maintained in a controlled environment with a light/dark cycle of 12 h, a temperature of 20 ± 2°C, and a humidity of 50 ± 2%. Male C57BL/6J mice were randomly assigned to 6 treatment groups: groups 1-2 received 0.9% saline, groups 3–6 were intraperitoneally (i.p.) injected with myriocin 0.3 mg/kg three times over 5 days (days 1, 3, and 5) [51], desipramine 20 mg/kg/day for 5 days, D609 10 mg/kg for 12 h before sacrifice, and NOE 10 mg/kg/day for 5 days, respectively. C57 mice of groups 2–6 were injected via tail vein with FITC-oxLDL (50 *μ*g/mouse) for 3 h before sacrifice, while mice of group 1 were injected with unlabeled oxLDL (50 *μ*g/mouse). Mice were euthanized, and the hearts, spleens, and livers were quickly frozen in liquid nitrogen. Frozen sections of hearts, spleens, and livers were prepared as described previously [49, 52]. The sections were stained with DAPI. Due to the existence of spontaneous fluorescence in aortas, we use the difference in fluorescent intensity between the subendothelial layer and the whole vessel to stand for FITC-oxLDL fluorescent intensity. For each group, 20 sections were analyzed, which represent 5 interval sections per aorta from 4 mice.

### 2.8. Isolation of Cavolin-1-Enriched Membrane Raft Fractions

Caveolae-enriched membrane fractions were prepared by a detergent-free purification, as described previously [41, 53]. To isolate membrane raft fractions from the cell membrane, HUVECs were lysed in 2 mL 500 mmol/L Na_2_CO_3_ containing protease inhibitor cocktail. Cell extracts were homogenized with 15 strokes through a 25-gauge needle followed by sonication for 15 s three times on ice. Detecting the concentration of protein in the homogenate made sure that each group has equal amounts of protein. The final volume was adjusted to 2 mL with MBS containing 25 mmol/L 2-(N-morpholino)ethanesulfonic acid and 0.15 mol/L NaCl, pH 6.5. Homogenates were adjusted with 2 mL 90% sucrose density gradient medium prepared in MBS to 45% and overlaid with discontinuous 4 mL 30% and 4 mL 5% sucrose in the MBS buffer containing 250 mmol/L Na_2_CO_3_. Samples were then centrifuged at 39,000 rpm for 18 h at 4°C in a SW 41 rotor (Beckman Instruments). A total of 12 fractions per 1 mL were collected carefully from top to bottom. For immunoblot analysis of membrane raft-associated proteins, these fractions were precipitated by 10% cold trichloroacetic acid and washed with cold acetone, air-dried. The protein pellets were then dissolved in an SDS-PAGE lysis buffer for western blot analysis.

### 2.9. Western Blotting

After cells were incubated with the inhibitors, respectively, for 12 h, caveolin-1 enriched membrane fractions were isolated as described above and the final samples were detected by western blotting. The protein samples were separated by SDS-PAGE gel and then electrotransferred to PVDF membranes. Subsequently, blots were subjected to immunostaining with antibodies against Caveolin-1 (Cell Signaling Technology, 1 : 800), Cavin-1 (ANBO, 1 : 500), and lectin-like oxLDL receptor (Lox-1, WuXi AppTec, 1 : 1000). After incubation for 1 h with a peroxidase-conjugated secondary antibody (Abbkine, 1 : 10000), bands were visualized by an ECL western blotting detection system (NDR, Israel). The band intensities were quantified using ImageJ software.

### 2.10. Statistical Analysis

All data are expressed as the mean ± SEM from at least three separate experiments. Significant differences between multiple groups were examined using ANOVA with Duncan's multiple-range testing. A value of *P* < 0.05 was considered significant.

## 3. Results

### 3.1. Endogenous Cellular Ceramide Production Is Regulated by Ceramide Metabolizing Enzyme Inhibitors

**Figure 1:**
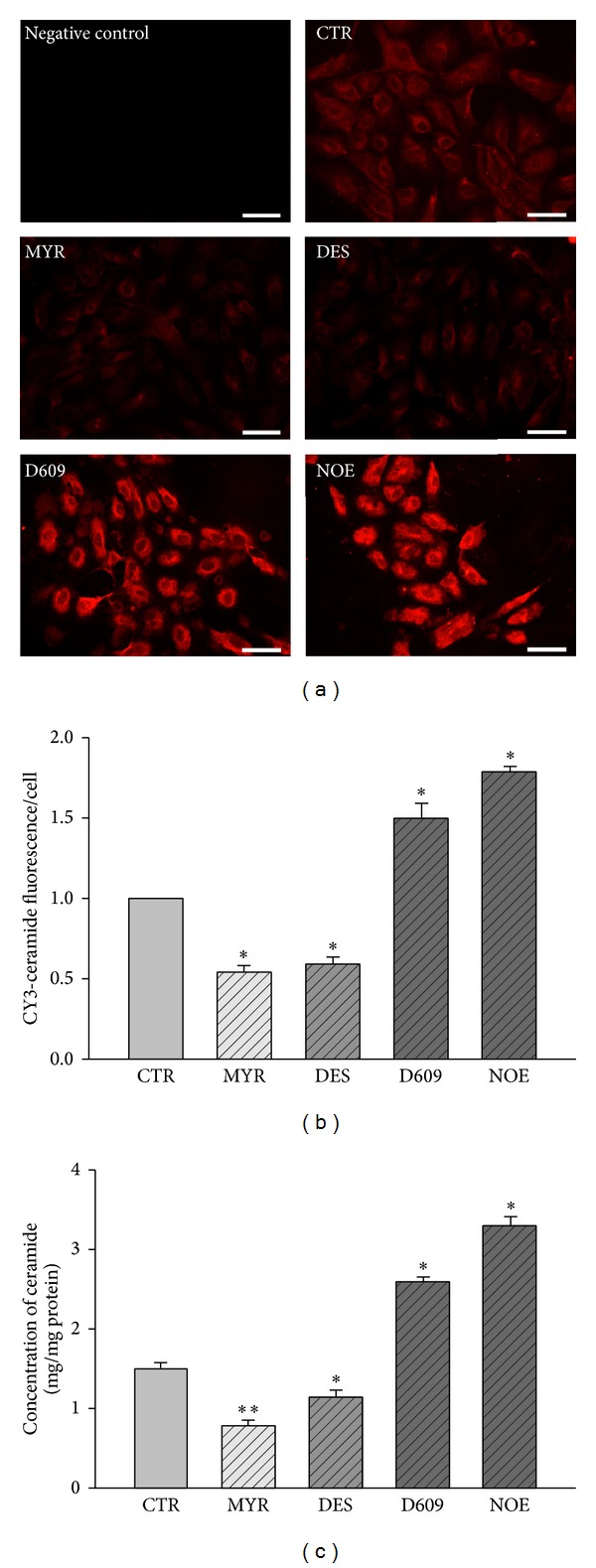
The effects of various inhibitors on ceramide concentration in HUVECs. HUVECs were incubated with 30 *μ*M MYR, 10 *μ*M DES, 30 *μ*M D609, or 10 *μ*M NOE for 12 h. Representative fluorescence microscopic images of ceramide (a) and quantification analysis (b) of immunofluorescent staining in HUVECs. Scale bars are equal to 50 *μ*m. “Negative control” group was processed in the absence of primary antibody. (c) Concentration of ceramides in HUVECs quantified by HPLC-MS analysis. *P < 0.05 versus control, *n* = 3.

To determine the effects of various inhibitors on ceramide metabolism, ceramide concentration was detected by two methods. The representative fluorescence microscopic images and semiquantitative results were shown in Figures 1(a) and 1(b). To further confirm the effects, we detected ceramides by HPLC/MS (Figure 1(c)). Results demonstrated that MYR and DES reduced ceramide concentration, while D609 and NOE increased ceramide concentration remarkably.

### 3.2. FITC-oxLDL Transcytosis across Endothelial Cell Monolayers

**Figure 2:**
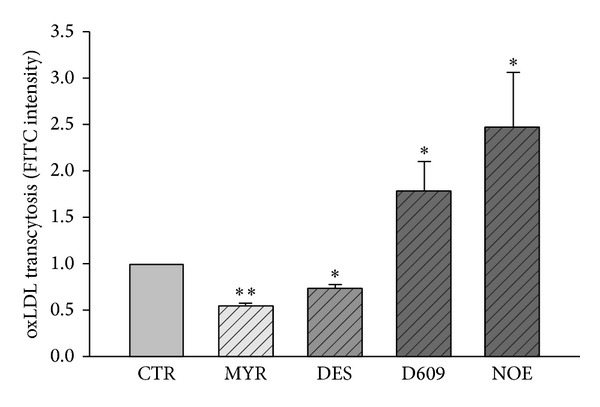
oxLDL transcytosis in the absence or presence of various inhibitors. HUVECs were incubated with 30 *μ*M MYR, 10 *μ*M DES, 30 *μ*M D609, or 10 *μ*M NOE for 12 h and FITC-oxLDL (50 *μ*g/mL) or oxLDL (300 *μ*g/mL) for 3 h. *P < 0.05, **P < 0.01 versus control, *n* = 4.

**Figure 3:**
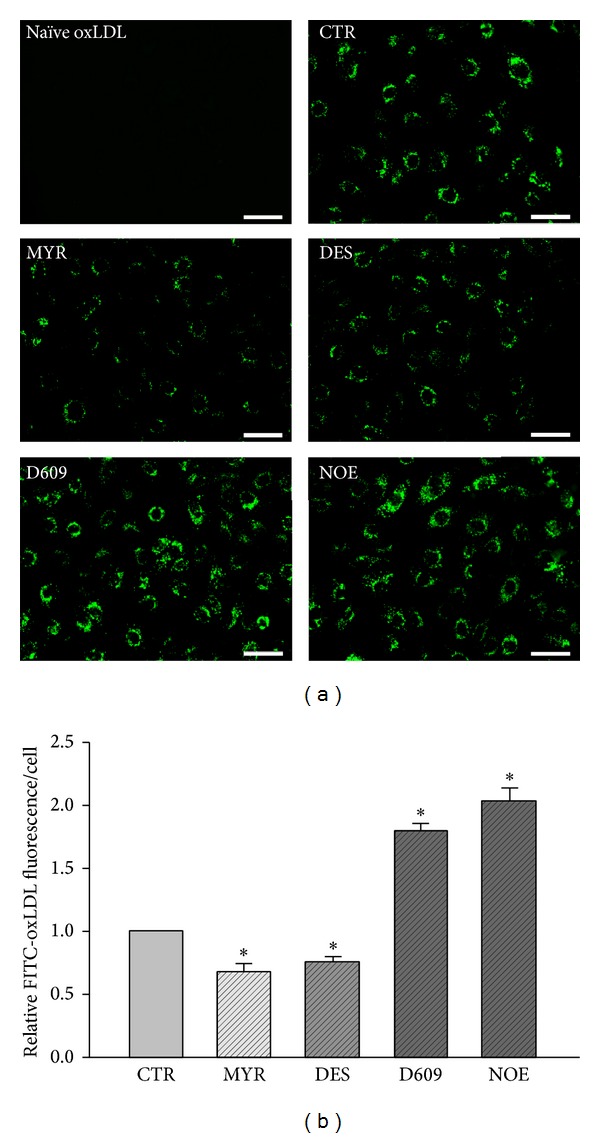
Fluorescence microscopic analysis of FITC-oxLDL uptake in HUVECs. HUVECs cultured on coverslips were pretreated with 30 *μ*M MYR, 10 *μ*M DES, 30 *μ*M D609, or 10 *μ*M NOE for 12 h, and oxLDL (50 *μ*g/mL) was added to incubate for 3 h. (a) Representative fluorescence microscopic images of FITC-oxLDL uptake of HUVECs. Scale bars are equal to 50 *μ*m. “Naïve oxLDL” group was incubated with unlabeled oxLDL. (b) Quantification analysis of FITC-oxLDL uptake in HUVECs. *P < 0.05 versus control, *n* = 3.

To determine whether the inhibitors alter the amount of oxLDL transport across HUVECs, we assayed the amount of oxLDL transcytosis across HUVECs. As shown in Figure 2, pretreatment with MYR or DES significantly decreased oxLDL transcytosis, while exposure to D609 or NOE significantly increased oxLDL transcytosis. These results were further confirmed by the observations of oxLDL uptake in cultured HUVECs. Since the oxLDL uptake by HUVECs is an intermediate phase of oxLDL transcytosis across HUVECs, it may also represent the amount of oxLDL transcytosis in a degree. As shown in Figures 3(a) and 3(b), fluorescence intensities in each individual cell were measured to reflect the amount of oxLDL uptake. It was found that the levels of oxLDL uptake were suppressed by MYR or DES, while elevated by D609 or NOE.

### 3.3. The Subendothelial Retention of oxLDL* In Vitro*

**Figure 4:**
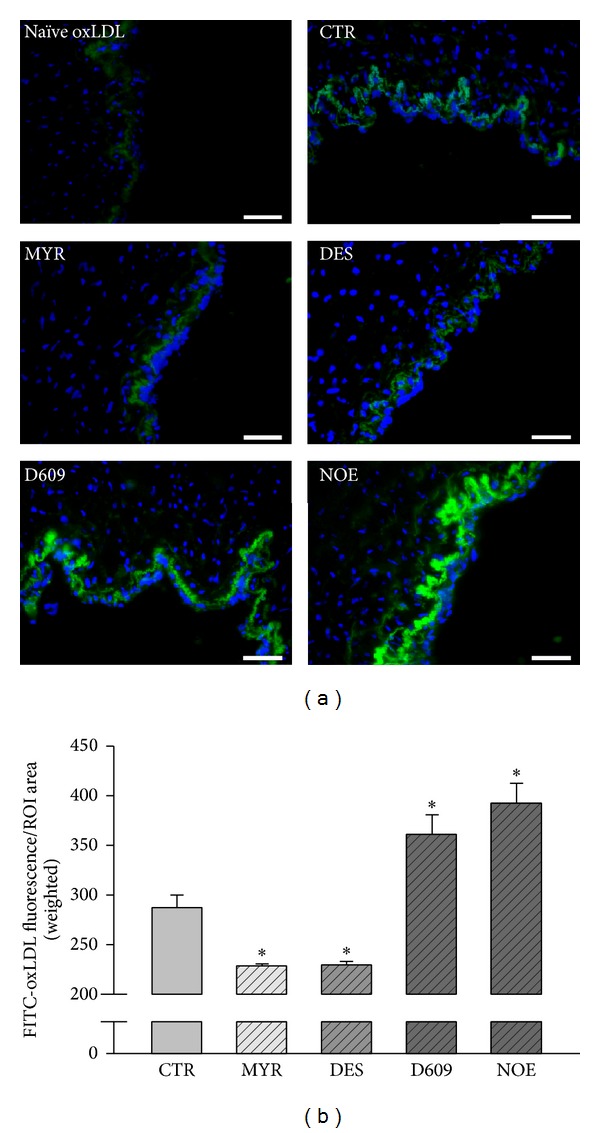
Fluorescence microscopic analysis of FITC-oxLDL (50 *μ*g/mL) retention in human umbilical venous walls. (a) Representative fluorescence microscopic images of FITC-oxLDL retention in human umbilical venous walls stimulated by PBS or various inhibitors. Scale bars are equal to 300 *μ*m. “Naïve oxLDL” group was incubated with unlabeled oxLDL. (b) Quantification analysis of FITC-oxLDL retention. *P < 0.05 versus control, *n* = 3.

An experiment was conducted to test whether subendothelial retention of oxLDL would alter in the presence of various inhibitors. As summarized in Figures 4(a) and 4(b), more FITC-oxLDL accumulated in the region above the basilar membrane after D609 or NOE stimulation. However, the accumulation of FITC-oxLDL was significantly decreased in the presence of MYR or DES.

### 3.4. The Subendothelial Retention of oxLDL* In Vivo*

**Figure 5:**
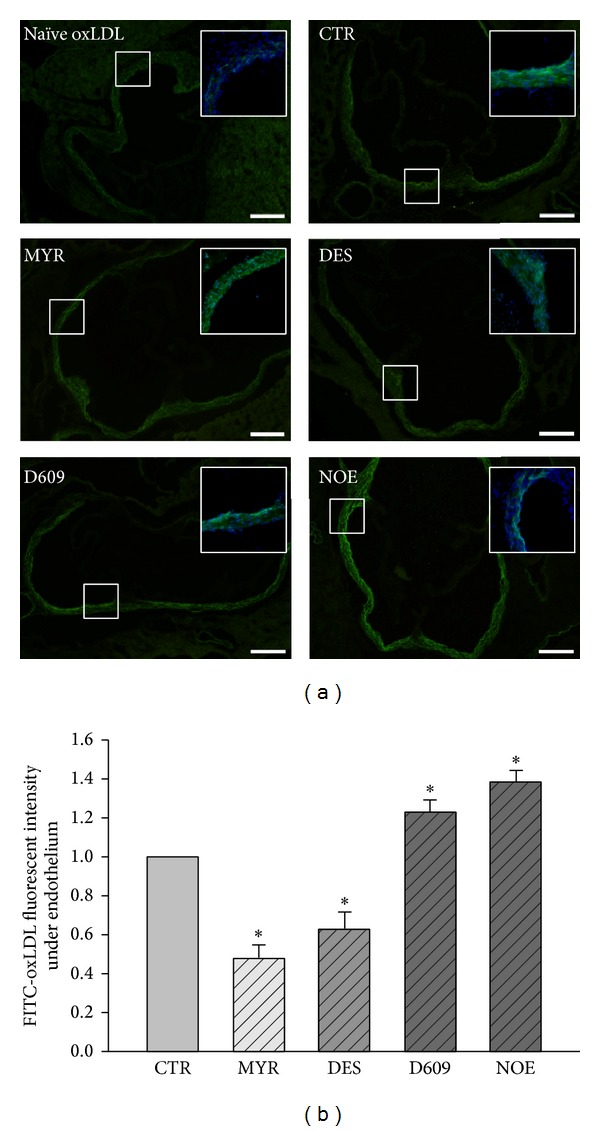
Fluorescence microscopic analysis of FITC-oxLDL retention in mouse aortic root. (a) Representative fluorescence microscopic images of aortic root sections of C57 mice after injection with FITC-oxLDL (50 *μ*g/mouse). Scale bars are equal to 500 *μ*m. Top-right panels, pictures with colocalization with DAPI. “Naïve oxLDL” group mice were injected with unlabeled oxLDL. (b) Quantification of FITC fluorescence intensity. *P < 0.05 versus control, *n* = 4.

To validate the oxLDL transcytosis and subendothelial retention, the endothelial fluorescence intensities in aortic roots from C57 mouse were detected. Compared to mice injected with unlabeled oxLDL, aortas from mice injected with FITC-oxLDL showed stronger fluorescence intensities located under endothelium (Figure 5(a)). Similar to that shown in Figure 4, the space under endothelium in aortic root of mice treated with inhibitors accumulated more or less fluorescence. It was noted that, aortas from MYR-/DES-treated mice accumulated very little fluorescence under endothelium, while aortas from D609-/NOE-treated mice showed much stronger fluorescence signal. Supplemental Figure 1 (see Supplementary Material online at: http://dx.doi.org/10.1155/2014/823071) showed no difference in fluorescence accumulation in spleens or livers of mice in separate groups.

### 3.5. The Expression of LRs Components Related to oxLDL Transcytosis

**Figure 6:**
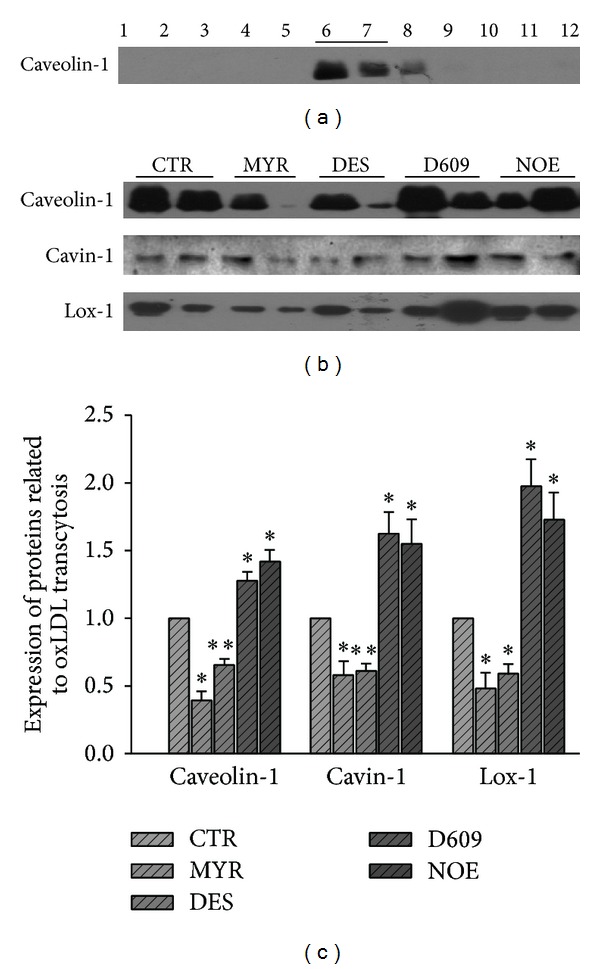
Expression of proteins related to oxLDL transcytosis in LRs in HUVECs. HUVECs were incubated with 30 *μ*M MYR, 10 *μ*M DES, 30 *μ*M D609, or 10 *μ*M NOE for 12 h and then LRs were isolated and detected with western blot. (a) Representative western blot showing the subcellular localization of the protein marker for LRs, caveolin-1. (b) Representative western blot showing the expression of proteins involved in oxLDL transcytosis in LRs, cavin-1 and Lox-1. (c) Quantitative analysis of the protein expression. *P < 0.05, **P < 0.01 versus control, *n* = 4.

Lipid rafts fractions were isolated as described before. Caveolin-1 enriched fractions (1 mL for each) were detected to determine LRs location (fractions 6 and 7) as shown in Figure 6(a). As shown in Figures 6(b) and 6(c), the expression of proteins involved in caveolae formation (caveolin-1 and cavin-1) as well as oxLDL receptor (Lox-1) could be regulated by inhibitors of ceramide related enzymes. Compared with control, MYR and DES significantly decreased the expressions of all proteins involved in oxLDL transport, while D609 and NOE increased the expressions.

## 4. Discussion

Ceramides are increasingly recognized to play essential roles in the pathogenesis of atherosclerosis [21, 54–58]. In the present study, for the first time, we demonstrated that endogenously produced ceramides in endothelial cells significantly contributed to the transcytosis of oxLDL across the endothelial cell barrier and facilitated the subendothelial retention of these oxidized lipids, further promoting the progression of atherosclerosis.

LDL* per se* is a spherical nanoparticle composed of lipid molecules surrounded by apoB100, which mediates the molecular recognition of LDL with its receptors. As compared to other types of lipoproteins, LDL contains more sphingolipids and cholesterol and has a property of resistance to detergent at low temperatures [7]. Previous reports have indicated elevated plasma sphingolipid levels in AS [59], pointing to the importance of ceramide in AS. But how ceramides affect atherogenesis remains to be further elucidated.

By immunostaining and HPLC-MS analysis, we first confirmed the inhibiting effects of the inhibitors of multiple enzymes involved in ceramide metabolism. We found that both* de novo* ceramide synthesis inhibitor, myriocin, and acid sphingomyelinase inhibitor, desipramine, could reduce the production of ceramide. However, ceramidase inhibitor, NOE, and sphingomyelin synthase inhibitor, D609, significantly upregulated the production of ceramide.

In an established* in vitro* model of oxLDL transcytosis across endothelial cell monolayer, we documented that myriocin and desipramine reduced the transcytosis of oxLDL; however, NOE and D609 significantly accelerated the transcytosis of oxLDL across endothelial cells. In this model, we used the difference of fluorescence between control and competitive inserts, which represent the total and paracellular transport of oxLDL, respectively, to reflect the transcytosis of oxLDL across endothelial cells. This method of detecting transcytosis had been validated by previous studies [47, 48]. The alterations of the transcytosis induced by various inhibitors in the present study are in consistence with the alterations of intracellular ceramide production, strongly indicating that the endogenously produced ceramides facilitate the transcytosis of oxLDL across endothelial cells. For oxLDL to be transcytosed across endothelial cells, the oxLDL particles must be endocytosed in the lumen side of the endothelial cells and then be transferred to the basolateral side and further be exocytosed to the subendothelial space. During this process, an intermediate state of transcytosis is that these particles had already been endocytosed into the cytosol but had not been released yet. Therefore, the intracellular concentration of oxLDL particles also reflects the activity of transcytosis. The decrease of oxLDL particles in myriocin and desipramine treated cells, whereas the increase of oxLDL particles in NOE and D609 treated endothelial cells, further supports the promoting roles of ceramide in regulating oxLDL transcytosis. In isolated vascular preparations, when the umbilical vascular segments were incubated with fluorescence-labeled oxLDL particles, these particles can be transported to the subendothelial space in the vessel walls. Myriocin and desipramine, which inhibited the production of intracellular ceramide, also reduced the subendothelial retention of oxLDL. Vice versa, NOE and D609, which upregulated the ceramide level, increased the subendothelial retention of oxLDL in vessel walls.

To further confirm these effects in* in vivo* level, we also conducted animal experiments. Similar to those observations in cultured endothelial cells and isolated vascular segments, the two inhibitors, myriocin and desipramine, which suppressed the production of ceramide, attenuated the accumulation of oxLDL in the mouse aorta walls. However, NOE and D609, which stimulated the ceramide production, enhanced the subendothelial retention of oxLDL in vessel walls.

After the oxidative modification of LDL, oxLDL is generated, in which the sphingomyelin hydrolysis rate is 5-6 times of the naive LDL [60]; ceramide levels in oxLDL particles in AS lesions are 10–50 times the ceramide levels in plasma natural LDL [61]. Our results are in consistence with several observations in previous reports. Devlin CM reported that ASM plays a very important role in the pathogenesis of AS. AS lesions in double gene-deficient mice from hybridization of ASM knockout* Asm* ^*−/−*^ mice and apolipoprotein E knockout* Apoe* ^*−/−*^ mice were significantly smaller than those in* Apoe* ^*−/−*^ mice [49]. Loidl et al. reported that oxidation of phospholipids of modified LDL activates intracellular ASM [62]. These observations also strongly support the essential role of ceramide in the formation of atherosclerosis. However, McGovern et al. reported that patients of Niemann-Pick disease types A and B with a deficiency in ASM activity had low HDL and elevated LDL in plasma and had high incidences of coronary atherosclerosis [63]. This controversy may be due to the different roles of ASM in the metabolism of lipid profiles in liver and in the lipoprotein retention in vascular wall. On one side, ASM appears to be essential to maintain the normal LDL and VLDL metabolism pathway. ASM deficiency in Niemann-Pick disease results in the elevation of plasma LDL and reduction of HDL, which are established risk factors of atherosclerosis. On the other side, ASM may directly promote the retention of lipoprotein particles into the subendothelial space of vascular wall and facilitate the progression of atherosclerosis. Our study focuses on the second aspect of ASM in vascular wall. This is very similar to the roles the LDL receptor (LDLR) plays in atherosclerosis. On one side, LDLR mediates the retention of LDL into the vascular wall and promotes the incidence of AS [64, 65]. On the other side,* Ldlr* ^*−/−*^ mice exhibit typical hypercholesterol and are more vulnerable to atherosclerosis [66, 67]. A recent paper by Li et al. suggested that the control of lysosome trafficking and fusion by ASM is essential to a normal autophagic flux in coronary arterial smooth muscle cells [68]. Basically, there is no doubt that ASM is beneficial under physiological conditions, but, in chronic pathological conditions such as atherosclerosis, the ASM activities are persistently upregulated and would in turn drive the progression of diseases [49, 69]. In this situation, inhibition of ASM activity may be the right strategy for therapy.

We also preliminarily studied the mechanism why ceramide contributed to the transcytosis of oxLDL. Basically, oxLDL transcytosis in endothelial cells is mediated by its receptor, Lox-1 [70, 71], as well as many other essential proteins involved in endocytosis or exocytosis, including the caveolae structure protein, caveolin-1 [71, 72], and caveolae associated protein, cavin-1 [72, 73]. Lox-1 and caveolin-1 are both residing in membrane raft domains of endothelial cells. Cavin-1 also binds to caveolin-1 to help maintain the integrity of caveolae. Since ceramides are much less polar than sphingomyelin, these hydrophobic lipids are more ready to fuse simultaneously and contribute to the integrity of membrane raft structures [30], which will facilitate the lipid raft-dependent transcytosis. We studied whether ceramide could alter the expressions of these oxLDL transcytosis-related proteins in membrane rafts. We found that the expressions of Lox-1, caveolin-1, and cavin-1 in membrane raft domains were also significantly regulated by ceramide metabolizing enzyme inhibitors. DES and MYR decreased the expression of Lox-1, caveolin-1, and cavin-1 in membrane rafts. Whereas NOE and D609 upregulated the expressions of these proteins in membrane rafts. Our observations partially explain the critical role of ceramide in the transcytosis of oxLDL across endothelial cells. Some previous studies have also shown that oxLDL induced lipid rafts clustering in human coronary arterial endothelial cells [74] and Lox-1 increased in lipid rafts after oxLDL treatment [53]. oxLDL may also affect ceramide production in lipid rafts [74, 75]. These observations imply that oxLDL* per se* may also elicit a signaling to facilitate its own transcytosis, which would form a feedback forward mechanism and amplify the transcytosis process.

Collectively, both the* in vitro* and* in vivo* evidences provided in the present study strongly point to a conclusion that oxidized LDL, oxLDL, is able to traffic across the endothelial barrier through transcytosis and these processes are highly regulated by intracellular ceramide. Endogenous ceramide significantly promotes the transcytosis of oxLDL across endothelial cells. Therefore, novel compounds designed to manipulate the metabolism of ceramide production through targeting related enzymes may provide novel strategies for the prevention or treatment of atherosclerosis-related disorders.

## Supplementary Material

As described in methods 2.7, frozen sections of spleens and livers from each group were prepared. There is no significant difference of fluorescence between groups.
