# Supplementary material for: Endogenous Ceramide Contributes to the Transcytosis of oxLDL across Endothelial Cells and Promotes Its Subendothelial Retention in Vascular Wall
Source: Oxid Med Cell Longev. 2014 Apr 10;2014:823071. doi: 10.1155/2014/823071 (PMC4003761; doi:10.1155/2014/823071)
Supplement: Supplementary file 1 — As described in methods 2.7, frozen sections of spleens and livers from each group were prepared. There is no significant difference of fluorescence between groups. [file 823071.f1.pdf]

# Supplementary Figure

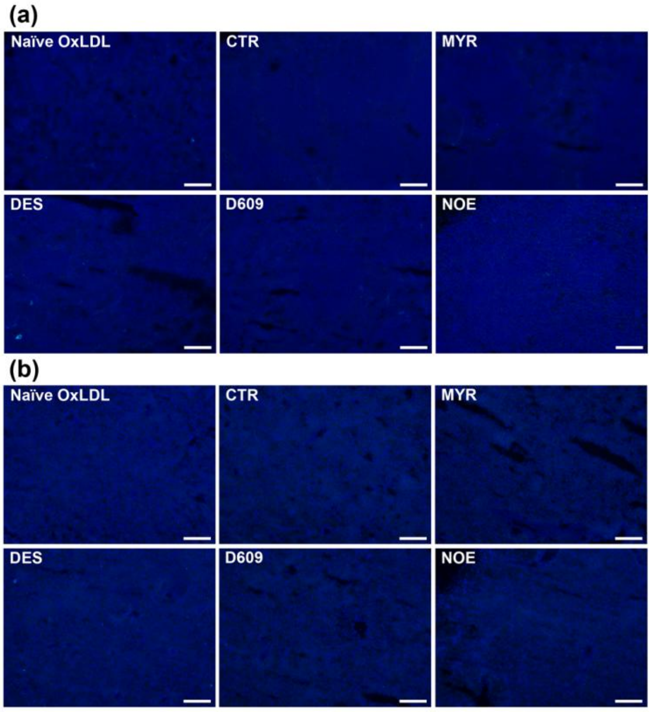


FIGURE S1: Fluorescence microscopic images of spleens and livers of C57 mice after injection with FITC-oxLDL (50 μg/mouse). "Naïve OxLDL" group mice were injected with unlabeled oxLDL. (a) Sections of spleen stained with DAPI. (b) Sections of liver stained with DAPI. Scale bars = 500 μm.
